# Giant paratubal cyst presenting as adnexal torsion: A case report

**DOI:** 10.1016/j.crwh.2020.e00222

**Published:** 2020-05-19

**Authors:** Filipa Alpendre, Isabel Pedrosa, Rita Silva, Serafim Batista, Paula Tapadinhas

**Affiliations:** a Centro Hospitalar e Universitário Lisboa Central, Lisboa, Portugal; b Centro Hospitalar do Baixo Vouga, Aveiro, Portugal; c Hospital Vila Franca de Xira, Vila Franca de Xira, Portugal

**Keywords:** Paratubal cyst, Paraovarian cyst, Ovarian torsion, Adnexal torsion, Case report

## Abstract

**Background:**

Paraovarian/paratubal cysts constitute about 10% of adnexal masses and are usually small and asymptomatic. A huge paratubal cyst complicated by adnexal torsion is a rare cause of acute low abdominal pain.

**Case Report:**

We report the case of an obese 31-year-old nulliparous woman who presented with a large pelvic cyst causing ovarian torsion. The size of the mass (~25 cm) caused pain, and obesity led to explorative laparotomy, which showed a huge central abdominal-pelvic cyst arising from the right adnexa. Cystectomy was technically impossible, so all the adnexa was removed. Pathologic diagnosis revealed a papillary serous cystadenoma with torsion of all structures.

**Conclusion:**

A giant paratubal cystadenoma is a rare condition and management is challenging. If there are clinical and imaging signs of torsion, it should be approached like any other adnexal mass and surgery should be urgent in order to avoid irremediable compromise of ovarian function.

## Highlights

• Paraovarian/paratubal cysts constitute about 10% of adnexal masses, and are usually small and asymptomatic.
• A huge paratubal cyst complicated by adnexal torsion is a rare cause of acute low abdominal pain.
• Suspected adnexal torsions require prompt surgical treatment in order to avoid compromises of ovarian function.

## 1 Introduction

A paraovarian/paratubal cyst is a fluid-filled sac that grows adjacent to the fallopian tube or the ovary [1]. The terms ‘paraovarian’ and ‘paratubal’ are used depending on the cyst's proximity to ovary or fallopian tube [2].

These cysts represent approximately 10% of all adnexal masses, are mostly benign and usually arise from paramesonephric and mesonephric remnants [1,3].

They are typically between 2 and 20 mm and noted as incidental findings during other pelvic surgeries [1,4]. Due to their size, they are usually asymptomatic, but when they reach larger sizes, they can lead to symptoms such as pelvic pain, or, more rarely, cause haemorrhage, rupture or torsion [5]. Malignant paraovarian tumours are very rare and benign paraovarian histological types include serous cystadenoma, papillary serous cystadenoma, serous cystadenofibroma, mucinous cystadenoma and endometroid cystadenoma [2].

We report a case of a giant paratubal cyst which presented as acute tubo-ovarian torsion.

## 2 Case Description

An obese 31-year-old nulliparous woman was admitted to the general emergency service with back pain that had started one week earlier and gradually become constant and increased in intensity. She was also vomiting but denied fever, dysuria, constipation, diarrhoea or anorexia. She had never been pregnant and had had amenorrhea for the past 7 years, due to polycystic ovary syndrome. Her body mass index was 39 kg/m^2^ (height 168 cm; weight 110 kg); she was taking no medication and had no other surgical or medical history to disclose.

During her physical exam, her vital signs were normal and abdominal examination revealed only tenderness in right quadrants, without guarding or rebound. Blood tests were performed and revealed a high white blood cell count (16,700 per mm^3^, 72% neutrophils), normal renal function and were negative for C-reactive protein. Urine analysis and pregnancy test were also negative.

**Fig. 1:**
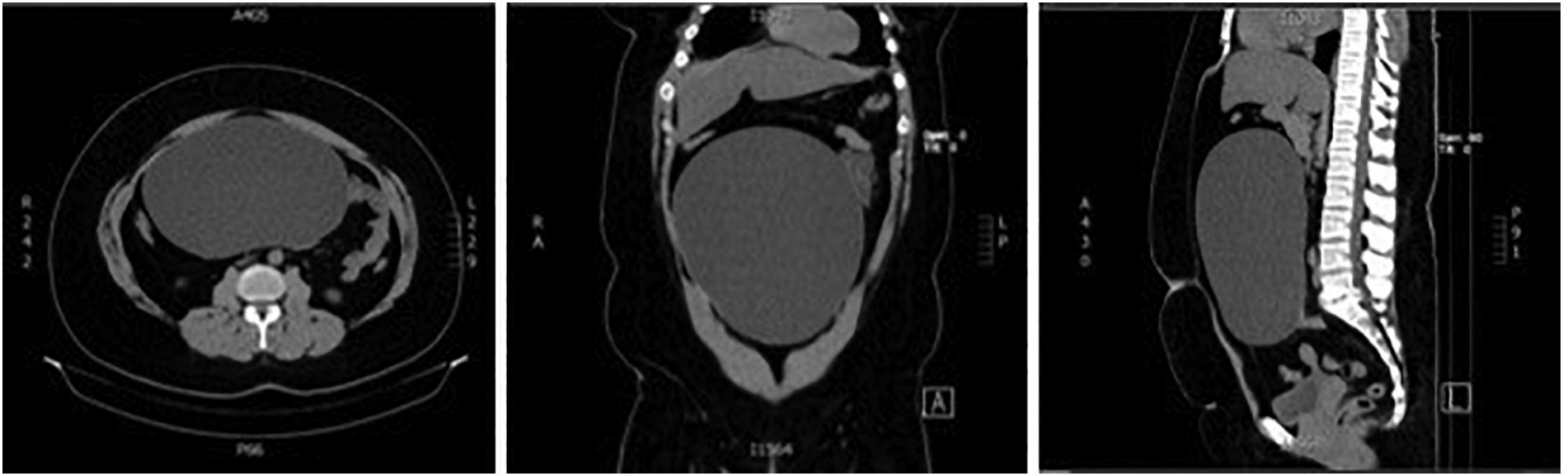
CT scan showing a giant central cyst.

Because her pain worsened, despite the administration of analgesic drugs, the patient underwent an abdominal and pelvic computed tomography (CT) scan, which showed a huge central abdominal-pelvic cyst arising from the right adnexa (Fig. 1). The cyst measured 25 cm × 20 cm × 14 cm and there was an adjacent enlarged oedematous ovary, highly suggestive of torsion. The left ovary, uterus and the appendix were normal and no free fluid in the abdomen and pelvis was observed.

She was then referred to the gynaecology department. Her gynaecologic exam was apparently normal but limited due to obesity. A pelvic ultrasound revealed a 20 cm × 20 cm thin-walled mass of the right adnexa, not clearly separate from it. Doppler examination showed that the right ovary was visible and had a polycystic ovarian morphology and apparent presence of vascular flow. The left ovary had the same polycystic pattern.

**Fig. 2:**
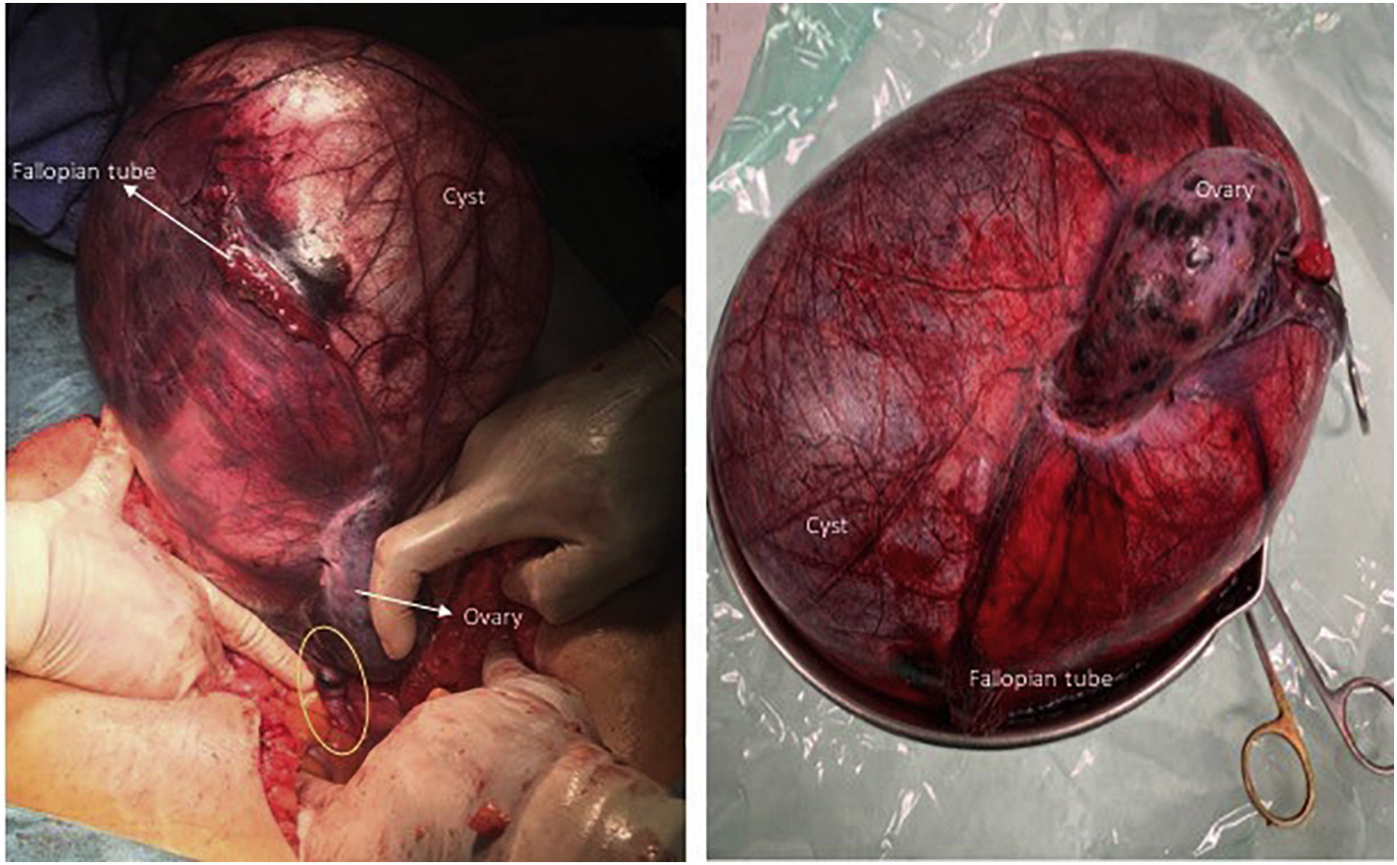
Intraoperative view of adnexal torsion: giant cyst, swollen and necrotic ovarian tissue, stretched fallopian tube and adnexal pedicle (yellow outline). The mass weighed 3727 g. (For interpretation of the references to colour in this figure legend, the reader is referred to the web version of this article.)

Her pain became acutely worse, which reinforced the hypothesis of adnexal torsion. Based on these findings and the increased pain, we decided to perform exploratory laparotomy. Intraoperatively, a giant (~25 cm) cystic median mass of apparent right adnexal origin was seen, occupying the entire pelvis up to the diaphragm. The cyst wall was intact, adhesion-free and swollen. Necrotic ovarian tissue was identified within the mass, but no cleavage planes were identified. The pedicle, which included the infundibulopelvic and utero-ovarian ligaments, was twisted twice around its stalk (Fig. 2). The left adnexa and the uterus looked normal.

The patient underwent resection of the mass without rupture. Frozen-section analysis was not available since the surgery was performed during the night. The final pathology report noted a paratubal serous papillary cystadenoma, an ovary with polycystic morphology and a normal fallopian tube. All fragments had congestion, oedema and haemorrhage, consistent with obstruction of the arteriovenous flow (torsion). The postoperative course was uneventful.

## 3 Discussion

Paratubal or paraovarian cysts are more commonly found in the third and fourth decades of life, but there are reports of cases in children, adolescents and postmenopausal women [4,[6], [7], [8], [9], [10]]. These cysts are mostly diagnosed incidentally during operations for other purposes. Few cause symptoms [7] and they rarely grow and have complications such as haemorrhage, rupture or torsion [10].

A paraovarian cyst is clinically similar to an ovarian cyst and imaging techniques can help with the diagnosis. Ultrasound is useful for detecting a round/oval cyst close to but separate from the ipsilateral ovary [8,11]. The split sign (separation of the cyst from the ovary during a endovaginal probe movement) also helps the diagnosis [8]. Nevertheless, diagnosis is difficult when these cysts are very close to the tube or ovary, especially if they are large. Differential diagnosis by ultrasound includes ovarian cyst, hydrosalpinx and peritoneal inclusion cyst [2,8].

Paraovarian/paratubal cysts are symptomatic when they become larger, but, even then, complications such as haemorrhage, torsion or rupture are rare [5]. These cysts have no pedicle, so, when they do twist, the ovary, the tube and the infundibulopelvic ligament are often involved [2,4,9]. The presentation of paraovarian/paratubal torsion is the same as that with an ovarian torsion, and includes intense lower abdominal pain and nausea and vomiting. More common conditions such as acute appendicitis, ruptured ovarian cyst, acute ureteric colic, ectopic pregnancy or pelvic inflammatory disease can manifest in the same way [10]. In this case, the large volume of the cyst did not allow its position, in relation to the ovary, to be clarified with certainty. CT was useful because our patient was obese and it permitted other causes of lower abdominal pain such as appendicitis to be excluded, and alerted us to signs of torsion.

Cysts larger than 10 cm and clinical suspicion of torsion require surgery [7]. Diagnosis should be quick in order to preserve long-term ovarian function [4] and treatment consists of detorsion and cystectomy to avoid recurrence [5].

Laparoscopy is the preferred approach for the diagnosis and treatment of adnexal torsion, but experience is required and size of the lesion is a limiting factor [5]. This nulliparous young woman had advantages in performing a less invasive surgery, but obesity and the huge mass in apparent torsion contributed to surgery being performed by laparotomy. Technical difficulty associated with trocar insertion, concerns regarding cyst rupture and the unavailability of an experienced laparoscopic surgeon supported our decision. Intraoperatively, since it was not possible to identify the origin of the huge cystic lesion with involvement of the ovary or to define cleavage plans, total excision of the lesion was performed, including ovary and tube. The histological report confirmed the diagnosis of papillary serous cystadenoma and torsion of all the structures.

## 4 Conclusion

Paraovarian/paratubal cysts are frequent but only in a few cases do they reach clinically relevant dimensions. Complications such as torsion and rupture are rare and are rarely diagnosed preoperatively. Differential diagnosis of acute abdominal pain must consider this condition and imaging can clarify the diagnosis and detect signs of acute complications, including torsion.

In the case of suspected adnexal torsion in young women, prompt surgical treatment is required in order to avoid irremediable compromise of ovarian function and fertility.
